# Overexpression of BubR1 Mitotic Checkpoint Protein Predicts Short Survival and Influences the Progression of Cholangiocarcinoma

**DOI:** 10.3390/biomedicines12071611

**Published:** 2024-07-19

**Authors:** Nongnapas Pokaew, Piya Prajumwongs, Kulthida Vaeteewoottacharn, Sopit Wongkham, Chawalit Pairojkul, Kanlayanee Sawanyawisuth

**Affiliations:** 1 Department of Biochemistry, Faculty of Medicine, Khon Kaen University, Khon Kaen 40002, Thailand; nongnapas.po@kkumail.com (N.P.); prajumwongspiya@gmail.com (P.P.); kulthidava@kku.ac.th (K.V.); sopit@kku.ac.th (S.W.); 2 Center for Translational Medicine, Faculty of Medicine, Khon Kaen University, Khon Kaen 40002, Thailand; 3 Department of Pathology, Faculty of Medicine, Khon Kaen University, Khon Kaen 40002, Thailand; chawalit-pjk2011@hotmail.com

**Keywords:** BubR1, BUB1B, mitotic checkpoint, cholangiocarcinoma

## Abstract

Budding Uninhibited by Benzimidazole-Related 1 (BubR1) or BUB1 Mitotic Checkpoint Serine/Threonine Kinase B (BUB1B) is an essential component of the spindle assembly checkpoint (SAC), which controls chromosome separation during mitosis. Overexpression of BubR1 has been associated with the progression of various cancers. This study demonstrated that high expression of BubR1 correlated with cholangiocarcinogenesis in a hamster cholangiocarcinoma (CCA) model and was associated with shorter survival in patients with CCA. Co-expression of BubR1 and MPS1, which is a SAC-related protein, indicated a shorter survival rate in patients with CCA. Knockdown of BubR1 expression by specific siRNA (siBubR1) significantly decreased cell proliferation and colony formation while inducing apoptosis in CCA cell lines. In addition, suppression of BubR1 inhibited migration and invasion abilities via epithelial–mesenchymal transition (EMT). A combination of siBubR1 and chemotherapeutic drugs showed synergistic effects in CCA cell lines. Taken together, this finding suggested that BubR1 had oncogenic functions, which influenced CCA progression. Suppression of BubR1 might be an alternative option for CCA treatment.

## 1. Introduction

Cholangiocarcinoma (CCA) is primary cancer that develops from bile duct epithelial cells and is highly prevalent in northeast Thailand because of an elevated incidence of *Opisthorchis viverrini* liver fluke infection, which causes chronic inflammation in bile duct cells and leads to CCA [1]. Patients with CCA are diagnosed with advanced-stage disease and poor prognosis [2]. CCA therapies include surgery and chemotherapy, which are ineffective at an advanced stage. A novel targeted therapy is required for an alternative treatment of CCA.

The spindle assembly checkpoint (SAC) is a cell cycle checkpoint that regulates chromosome separation by delaying sister chromatid separation from metaphase to anaphase until all kinetochores are properly attached to the mitotic spindle [3]. SAC protein core components include MAD1, MAD2, BUB1, BUB3, BUB1B, and MPS1 [4]. In daughter cells, an abnormal SAC causes chromosomal segregation abnormalities, leading to unequal chromosome numbers and aneuploidy. The accumulation of aneuploidy in normal cells could lead to neoplastic transformation [5]. Budding Uninhibited by Benzimidazole-Related 1 (BubR1) or Budding Uninhibited by Benzimidazoles 1 Homolog Beta (BUB1B) is important for SAC signaling and the stable attachment of kinetochores to spindle microtubules [6]. BubR1 overexpression has been reported in several human cancers and is involved in neoplastic progression, including the cell survival and metastatic potential of liver, lung, and thyroid cancer, including CCA [7,8,9,10].

In this study, we analyzed BubR1 expression in CCA from public databases. The significance of BubR1 expression was also examined in hamster and human CCA tissues from our biobank. The functional roles of BubR1 in the progression of CCA, including cell proliferation, migration, and invasion, were explored in CCA cell lines.

## 2. Materials and Methods

### 2.1. Hamster Tissues and Tissues from CCA Patients

Paraffin-embedded liver tissues were obtained from a CCA hamster model with *Opisthorchis viverrini* (Ov) that was separated into four groups, including (1) non-treated, (2) Ov-infected, (3) N-nitrosodimethylamine (NDMA; 12.5 ppm in water ad libitum)-treated, and (4) CCA (Ov + NDMA) groups. The liver tissues were collected one, three, and six months after treatment. The study protocol was approved by the Ethics Committee for Animal Research, Khon Kaen University (AEMDKKU 001/2558). Human tissues from patients with CCA were obtained from the Cholangiocarcinoma Research Institute, Faculty of Medicine, Khon Kean University. The research protocols were approved by the Ethics Committee for Human Research, Khon Kaen University (HE641574). All samples used were the same set used in our previous study [11].

### 2.2. Immunohistochemistry

Tissue sections were deparaffinized in xylene and rehydrated in different concentrations of ethanol at 100%, 90%, and 70%, respectively. Tris–EDTA buffer at pH 9 was used in an autoclave for antigen retrieval. Neutralization of endogenous peroxidase was performed using 0.3% H_2_O_2_ in methanol, followed by the blockage of non-specific binding with 1% skimmed milk. To detect BubR1 expression, a section was incubated with 1:300 anti-BubR1 (Abcam, Cambridge, UK) overnight at 4 °C, and then incubated with a secondary antibody (EnVision + System-HRP-Labelled polymer anti-Mouse, Dako, Glostrup Kommune, Denmark). Next, the section slices were stained with a 3, 3-diaminobenzidine substrate solution and counterstained with Mayer’s hematoxylin. BubR1 expression was examined using H-scores, which have a range of 0–300 based on intensity. The intensity was considered to be 0–3, as follows: 0, absent; 1+, weak; 2+, moderate; and 3+, strong [12]. H-score = [1 × (% of 1+ cells)] + [2 × (% of 2+ cells)] + [3 × (% of 3+ cells)].

### 2.3. Datasets of BubR1 Expression

For the gene expression profile of BubR1 (BUB1B) in CCA tissues (n = 36) and normal adjacent tissues (n = 9), the overall survival of patients with CCA divided into high (n = 18) and low (n = 18) groups was retrieved from The Cancer Genome Atlas (TCGA) databases and analyzed using the Gene Expression Profiling Interactive Analysis (GEPIA) web-based tool (http://gepia.cancer-pku.cn/ accessed on 15 July 2024). Gene Expression Omnibus (GEO) databases (https://www.ncbi.nlm.nih.gov/geo/ accessed on 15 July 2024) were used; the accession number is GSE76297 based on GPL17586 Affymetrix Human Transcriptome Array 2.0. The correlation between BubR1 and MPS1 expression was analyzed using GraphPad Prism^®^ 9.0 (GraphPad software, Inc., San Diego, CA, USA).

### 2.4. Human CCA Cell Lines

KKU-055 and KKU-213A CCA cell lines [13] were purchased from JCRB Cell Bank (Osaka, Japan) via the Cholangiocarcinoma Research Institute, and cultured in Dulbecco’s Modified Eagle’s Medium (DMEM) with 10% fetal bovine serum (FBS). Cells were cultured at 37 °C in a 5% CO_2_ humidified incubator.

### 2.5. Transient BubR1 Knockdown by siRNA

KKU-055 and KKU-213A cell lines were transfected with two pairs of siRNAs specific to the human BubR1 sequence as follows: #1 sense stand 5′-AAC AAT ACT CTT CAG CAG CAG-3′ and anti-sense stand 5′-CTG CTG CTG AAG AGT ATT GTT-3′, and #2 sense stand 5′-GAG CAG AAA TGC AGA AAC ATT-3′ and anti-sense stand 5′-TGT TTC TGC ATT TCT GCT CTT-3′ were used to study functional roles in CCA progression. A CCA cell was transfected with siRNA or a scramble control (sense stand 5′-TTC TCC GAA CGT GTC ACG TdTdT and anti-sense stand 5′-ACG TGA CAC GTT CGG AGA AdTdT) using Lipofectamine 2000 reagent (Invitrogen, Carlsbad, CA, USA). After transfection, the cell was harvested for further analysis.

### 2.6. Western Blot

Protein was extracted with Radio Immuno-Precipitation Assay (RIPA) Buffer (50 mM Tris-HCl, pH 7.4, 1% NP-40, 150 mM NaCl, 0.1 sodium dodecyl sulfate (SDS), 1 mM ethylenediaminetetraacetic (EDTA), and 1% sodium deoxycholic acid) containing phosphatase and protease cocktail inhibitors (EMD Millipore, Burlington, MA, USA). The Western blot was modified from that previously described [14]. Specific antibodies were used to identify proteins, including BubR1 (1:1000; Abcam, UK), ZO-1, Slug, Mcl-1, caspase 9, cleaved caspase 9, PRAP, cleaved PARP (1:1000), vimentin (1:500) (Cell Signaling Technology, Danvers, MA, USA), and GAPDH (1:5000; EMD Millipore, Darmstadt, Germany). The intensity of a protein band was analyzed using NIH ImageJ 1.52p (National Institutes of Health, Bethesda, MD, USA).

### 2.7. Cell Viability Assay

KKU-055 and KKU-213A cell lines were cultured in a 35 mm culture dish overnight and treated with 50 pmole of scramble and siBubR1 silencers for 24 h. The treated CCA cells were detached and seeded to a 96-well plate, with 2000 cells per well. Cell proliferation was determined at 24, 48, 72, and 96 h after transfection using MTT (3-(4,5-dimethylthiazolyl-2)-2,5-diphenyltetrazolium bromide) incubated for 4 h. A formazan complex was dissolved in isopropanol with 0.08N HCl, and cell viability was measured via absorbance at 540 nm.

### 2.8. Clonogenic Assay

CCA cell lines were cultured in a 35 mm culture dish overnight and treated with 50 pmole of scramble and siBubR1 silencers for 24 h. The treated CCA cells were detached and seeded to a 24-well plate, with 100 cells per well, and incubated at 37 °C in a humidified 5% CO_2_ incubator for 7 days for KKU-213A cells and 10 days for KKU-055 cells. Colonies were fixed in 4% paraformaldehyde and stained with 0.5% crystal violet.

### 2.9. Cell Cycle Analysis

CCA cells (2 × 10^5^ cells) were seeded into a 6 cm cell culture dish and treated with siBubR1 for 48 h. Cells were fixed in 70% ethanol at 4 °C overnight and stained with 50 μg/mL propidium iodide (PI). DNA content was analyzed using a FACSCanto II flow cytometer. Data analysis was performed using FlowJo 10.6.1 software.

### 2.10. Cell Migration and Invasion Assay

CCA cells (5 × 10^4^ cells) in serum-free DMEM were added to an insert (Transwell^®^ pore size 8 µm, Corning Incorporated, Corning, NY, USA). For the invasion assay, the insert was coated with 0.4 mg/mL of Matrigel overnight, which was pipetted out before seeding cells. The bottom chamber contained DMEM with 10% FBS. After incubation at 37 °C in a humidified 5% CO_2_ incubator, the migrated time was stopped for 24 and 12 h in KKU-055 cells and KKU-213A cells, and the invaded time was stopped for 35 and 6 h in KKU-055 cells and KKU-213A cells, respectively.

### 2.11. Combination Index Analysis

CCA cell lines were treated with siBubR1 or scramble control for 24 h, and then treated with different concentrations of gemcitabine, cisplatin, and 5-fluorouracil for 48 h. Cell viability was determined by an MTT assay. Compu-Syn 2.0 software (MIT, Cambridge, MA, USA) was used to calculate the combination indices (CI) and fraction affected (Fa) values. A CI >1 was defined as an antagonist effect; a CI = 1 was defined as an additive effect; and a CI < 1 was defined as a synergistic effect [15]. The dose reduction index (DRI) determined how many folds of dose reduction were allowed for each drug in synergistic combinations.

### 2.12. Statistical Analysis

The statistical significance of differences observed between the control group and the experimental groups was determined using a Student’s *t*-test. A value of *p* < 0.05 was considered significant. Values were presented as the mean ± SD. All data were analyzed by GraphPad Prism^®^ 9 software (GraphPad software, Inc., San Diego, CA, USA) and SPSS 17.0 software (SPSS, Chicago, IL, USA).

## 3. Results

### 3.1. BubR1 Expression Was Up-Regulated during Carcinogenesis in CCA Hamster Tissues

**Figure 1:**
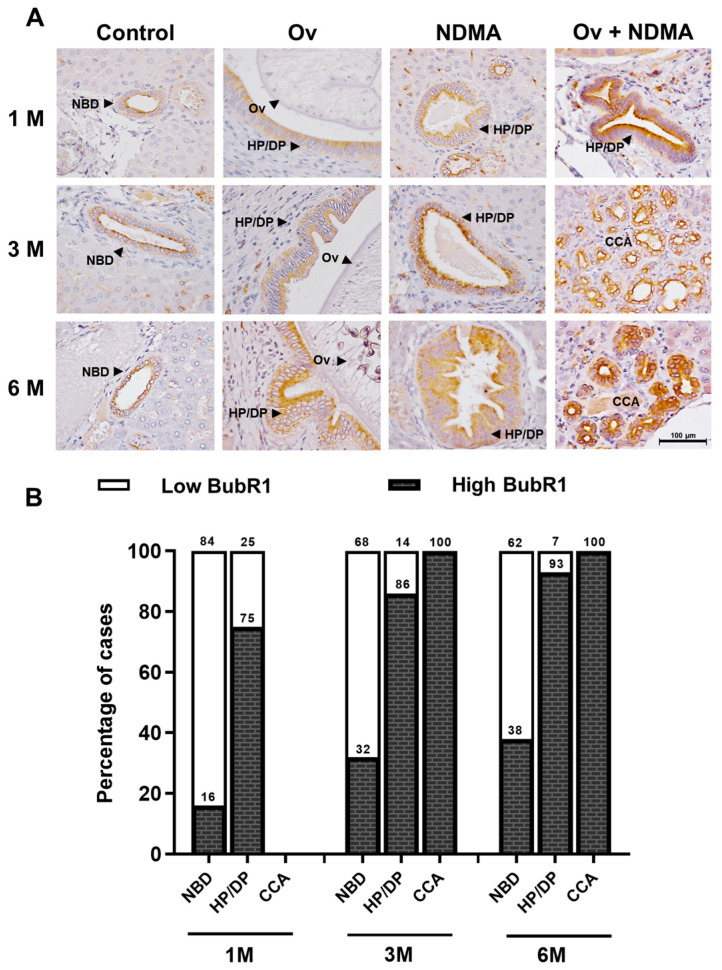
BubR1 is related to cholangiocarcinogenesis in a hamster model. (**A**) BubR1 was stained using immunohistochemistry (IHC) in the liver tissues of hamsters in four groups at one, three, and six months after treatment: control, N-nitrosodimethylamine (NDMA)-treated, Ov-infected, and a combination of NDMA + Ov. (**B**) A comparison of BubR1 expression between NBD and pathological bile ducts (HP/DP and CCA). The samples were divided into low-BubR1 and high-BubR1 groups, using a cutoff value of a median H-score of 133. NBD, normal bile duct; HP/DP, hyperplasia/dysplasia; CCA, cholangiocarcinoma; Ov, *Opisthorchis viverrini*.

We used immunohistochemistry to determine the correlation between BubR1 expression and cholangiocarcinogenesis in CCA hamster tissues. The result showed BubR1 was slightly expressed in normal bile ducts in the control group. The Ov-infected and NDMA-treated groups showed BubR1 expression gradually increased in hyperplasia/dysplasia (HP/DP) found at every time point. Likewise, BubR1 expression in CCA was found at 3 and 6 months. Whereas in Ov+NDMA-treated groups, BubR1 was highly expressed in HP/DP found at every time point and was highly expressed at 3 and 6 months of CCA. (Figure 1A). The percentage of BubR1-positive cases in HP/DP gradually increased with time depending on the treatment group and was highly expressed in CCA (Figure 1B). At three and six months, CCA had developed, and all tissues were BubR1-positive (Table 1). These results indicate that BubR1 could be detected early in precancerous CCA. This is the first report of BubR1 expression being up-regulated during cholangiocarcinoma in a hamster model.

### 3.2. BubR1 Was Overexpressed in CCA and Associated with the Short Survival of Patients with CCA

**Figure 2:**
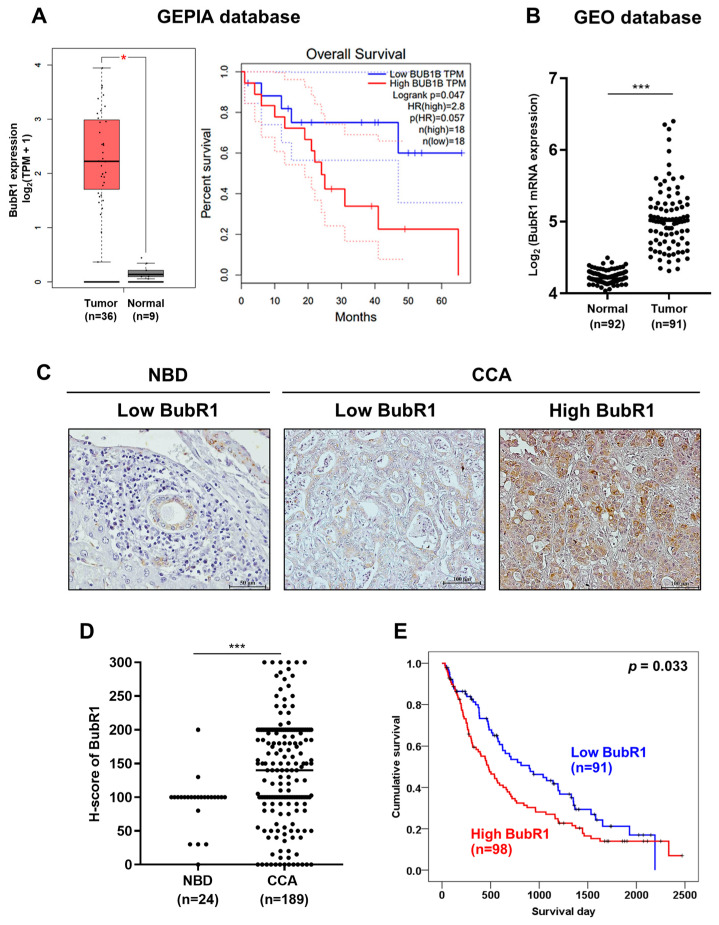
BubR1 was overexpressed in human CCA tissue. (**A**) BubR1 transcript levels in 36 human CCA tissues and nine normal bile ducts (NBDs) are shown in a box plot; TPM or transcripts per million. * *p* < 0.05. For a comparison of overall survival and BubR1 expression using Kaplan–Meier analysis, information was taken from the GEPIA database. (**B**) The distribution of BubR1 transcript levels in 183 human patients with CCA was categorized into normal (n = 92) and tumor groups (n = 91), using the median value of BubR1 expression (normal 4.2, tumor 5.0). Data were retrieved from the GEO database, *** *p* < 0.001. (**C**) Immunohistochemistry staining of BubR1 in human CCA tissues (n = 189). Low expression of BubR1 in the normal bile duct (NBD); low and high expression of BubR1 in the tumor area. (**D**) Distribution of BubR1 expression in 24 NBDs and 189 CCA tissues. The median H-score of NBDs was 100, and for CCA was 140, *** *p* < 0.001. (**E**) Kaplan–Meier analysis of the correlation between BubR1 expression and overall survival. A high expression of BubR1 (n = 98) had significantly shorter overall survival than that of BubR1 low expression (n = 91).

To investigate the level of BubR1 expression in CCA and normal tissues, data were retrieved from TCGA and GEO (GSE76297) datasets. Box plots were plotted for elucidation of the expression level of BubR1 genes in tissues from patients with CCA compared to normal tissues. The results showed that BubR1 mRNA expression was significantly higher in CCA cases (n = 36) when compared to normal adjacent tissues (n = 9), as shown in Figure 2A (*p* < 0.01). Patients with CCA were classified into high- and low-expression groups based on the median BubR1 level, and patients with CCA with high expression of BubR1 had a significantly shorter survival time than those with low expression (log-rank, *p* = 0.047). Additionally, results from the GEO dataset showed BubR1 expression was significantly higher in tumor tissues (n = 92) than in normal tissues (n = 91), as shown in Figure 2B (*p* < 0.001). Moreover, we performed immunohistochemistry (IHC) staining on tissue microarrays of 189 patients with CCA. BubR1 protein expression was mainly higher in CCA tissues but lower in normal bile ducts (Figure 2C). For the distribution of H-scores of BubR1, we set a cutoff value to separate low and high expression levels of BubR1 using the median H-score of 140 calculated from 189 CCA tissues, while 24 normal bile ducts (NBDs) showed a significantly lower median H-score of 100 (*p* < 0.001; Figure 2D). The survival time of patients with CCA with a high expression of BubR1 (98/189 cases, 51.9%) was significantly shorter than those with low BubR1 expression (91/189 cases, 48.1%; *p* < 0.05; Figure 2E). We performed univariate and multivariate analyses to determine whether BubR1 expression could be used to predict the prognosis of patients with CCA. The multivariate analysis revealed that high BubR1 expression was independently associated with short survival; the hazard ratio (HR) was 1.568 (95% CI 1.075–2.287) compared to the low BubR1 expression group, as shown in Table 2 (*p* = 0.02), which indicates that BubR1 is an independent prognostic factor for the short survival of patients with CCA.

### 3.3. Co-Expression of BubR1 and MPS1 Was Associated with the Short Survival of Patients with CCA

**Figure 3:**
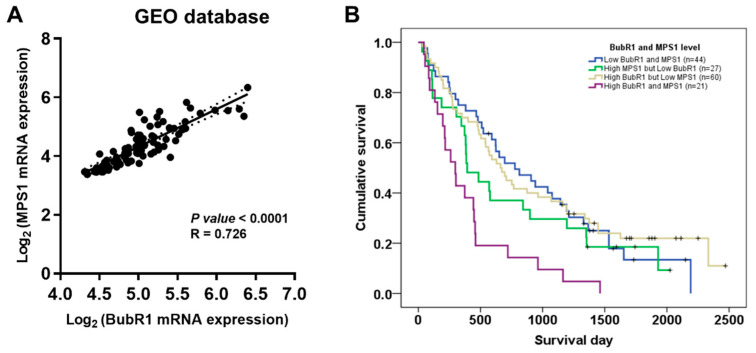
Association between BubR1 and MPS1 in CCA. (**A**) The correlation between the mRNA expression of BubR1 and MPS1 in CCA (n = 91). Data were retrieved from the GEO database (GSE76297). The solid line indicates mean mRNA expression, whereas the dotted line shows the standard deviation of mRNA expression. (**B**) A Kaplan–Meier plot of 152 patients with CCA who expressed high levels of both BubR1 and MPS1 (purple line) versus patients who expressed high BubR1 but low MPS1 (yellow line), high MPS1 but low BubR1 (green line), and low levels of both BubR1 and MPS1 (blue line).

Our previous study showed that high expression of MPS1 was an independent prognostic factor for the short survival of patients with CCA [11]. To investigate the association between BubR1 and MPS1, we analyzed GEO (GSE76297) datasets. There was a significant association between BubR1 expression and MPS1 expression at mRNA levels (Figure 3A). To further analyze the stronger evidence of prognostic factors for the short survival of patients with CCA, we combined the immunohistochemistry data of patients with CCA to analyze survival time and performed cox regression analysis that divided the expression of two molecules into four groups as follows: high BubR1 and MPS1 (21/152 cases, 14%), high BubR1 but low MPS1 (60/152 cases, 39%), high MPS1 but low BubR1 (27/152 cases, 18%), and low BubR1 and MPS1 (44/152 cases, 29%). The median survival time of patients with high BubR1 and MPS1 expression was significantly shorter than those with low BubR1 and MPS1 expression (Figure 3B, *p* < 0.001). Cox regression analysis was performed to determine whether BubR1 and MPS1 expression indicate a risk factor for short survival in patients with CCA. Multivariate analysis revealed that the hazards ratio (HR) of patients who were classified as BubR1 and MPS1 high expression was 2.778 (95% CI 1.578–4.891) compared to BubR1 and MPS1 low expression cases, which had a higher ratio than either BubR1 (Table 3) or MPS1 high expression [11]. This result indicates that co-expression of BubR1 and MPS1 could be an independent prognostic factor for the short survival of patients with CCA.

### 3.4. Suppression of BubR1 by siRNA Reduced Cell Proliferation, Colony Formation and Induced a Sub-G1 Population in CCA Cell Lines

**Figure 4:**
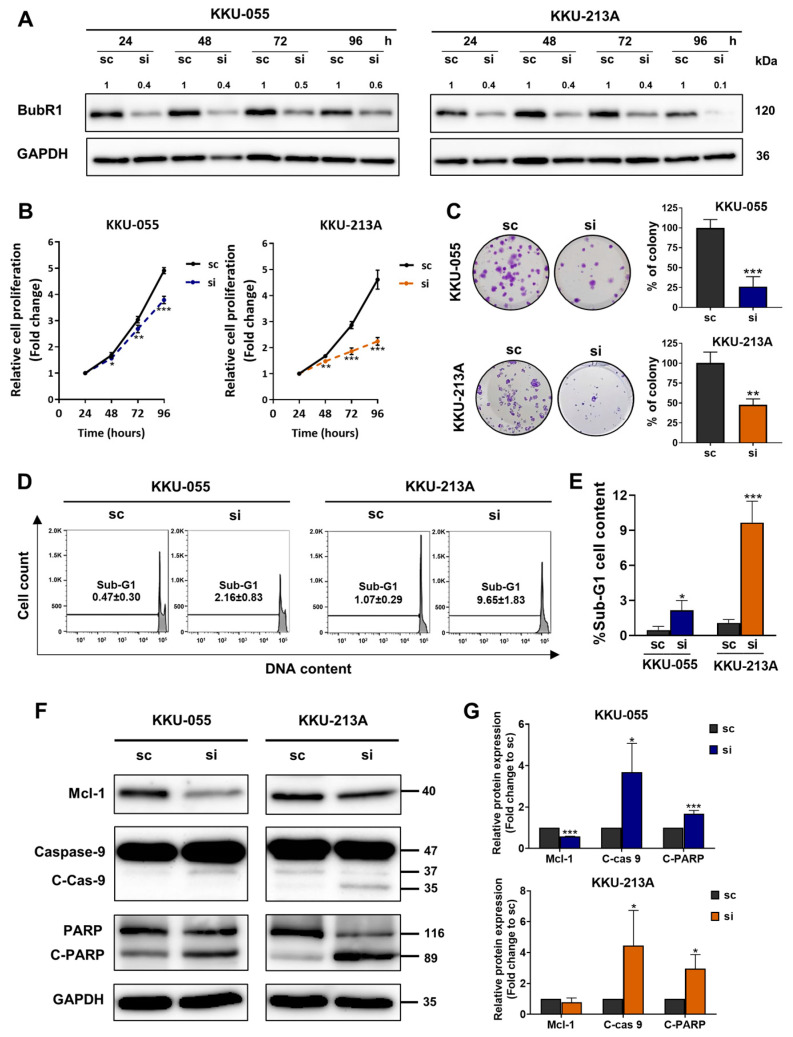
Suppression of BubR1 expression reduced cell proliferation, colony formation, and induced a sub-G1 population in a CCA cell line. (**A**) Representative Western blotting analysis of BubR1 expression after knockdown of BubR1 by siRNA at 24 to 96 h. (**B**) The cell proliferation rate was determined at 24 to 96 h using MTT and (**C**) clonogenic assays show the percentage of colonies after BubR1 knockdown in CCA cell lines. (**D**,**E**) Flow cytometry histograms demonstrate a sub-G1 population and a percentage of sub-G1 cell content in siBubR1-treated cells at 48 h. (**F**) Representative Western blotting analysis of Mcl-1, caspase 9, cleaved caspase 9, PARP, and cleaved PARP expression after knockdown of BubR1 at 48 h. (**G**) The bar graph represents the fold-change expression quantified using GAPDH, and the scramble (sc) control was set to one. The results (mean ± SD) are averages from three independent experiments; * *p* < 0.05, ** *p* < 0.01 and *** *p* < 0.001.

The roles of BubR1 in the progression of CCA were investigated in KKU-055 and KKU-213A CCA cell lines. BubR1 expression was transiently suppressed by siRNA for 24, 48, 72, and 96 h in CCA cell lines. Western blot results indicated that in both CCA cell lines BubR1 expression was successfully suppressed by the BubR1-specific siRNA (siBubR1) from 24 to 96 h (Figure 4A). To explore the role of BubR1 on cell proliferation, BubR1 was knocked down for 24, 48, 72, and 96 h with siBubR1, and then cell proliferation was determined by an MTT assay. Suppression of BubR1 for 48, 72, and 96 h showed significantly reduced relative cell proliferation in KKU-055 and KKU-213A cell lines (Figure 4B). The long-term effect of BubR1 suppression on colony formation from a single cell was investigated by a clonogenic assay. CCA cell lines were suppressed by siBubR1 and further cultured for 7 days (KKU-213A) or 10 days (KKU-055). The result showed that the percentage of the colony in the siBubR1 group was significantly lower (26.3% in KKU-055, 47.6% in KKU-213A) than in the scramble group (100%), as shown in Figure 4C. Cell cycle analysis showed that exposure to siBubR1 resulted in an increase in the number of apoptotic cells (sub-G1 population). After siBubR1 treatment, the % sub-G1 was 2.16 ± 0.83 in KKU-055 cells and 9.65 ± 1.83 in KKU-213A cells, as shown in Figure 4D,E. We examined the levels of protein expression for those involved in apoptosis. The results showed that knockdown of BubR1 significantly increased cleavage forms of caspase 9 and poly-adenosine diphosphate-ribose polymerase (PARP). Moreover, Myeloid cell leukemia-1 (Mcl-1), which is an anti-apoptotic protein, was decreased in the siBubR1 group (Figure 4F,G). This suggests that knockdown of BubR1 enhanced apoptotic cell death in CCA cell lines.

### 3.5. Knockdown of BubR1 Inhibited Cell Migration and Invasion of CCA Cell Lines

**Figure 5:**
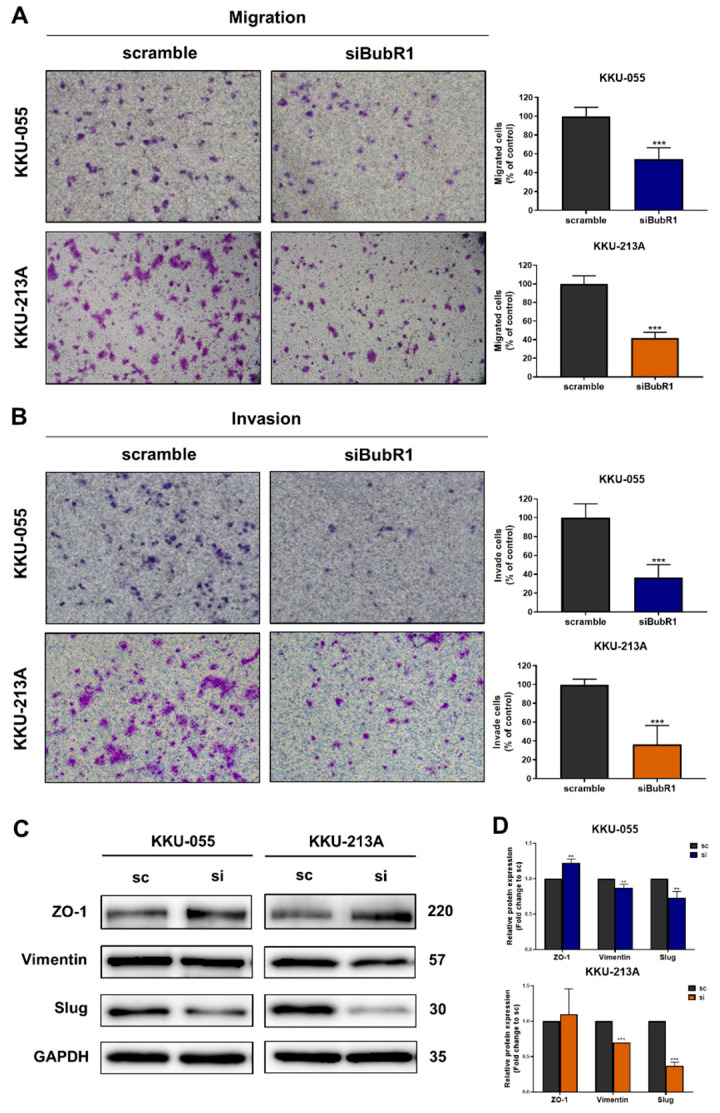
Knockdown of BubR1 inhibited cell migration and invasion of CCA cell lines. A Boyden chamber assay shows the percentage of (**A**) migrated and (**B**) invaded cells compared with those of scramble cells (100%). BubR1 regulated CCA cell migration and invasion abilities via the EMT process. (**C**) Representative Western blotting analysis of ZO-1, vimentin, and slug expression after knockdown of BubR1 at 24 h. (**D**) The bar graph represents the fold-change expression quantified using GAPDH, and the scramble (sc) control was set to one. The results (mean ± SD) are averages from three independent experiments; ** *p* < 0.01, *** *p* < 0.001.

To investigate roles of BubR1 on migration and invasion of CCA cell lines, a Boyden chamber assay was performed to study the migration and invasion abilities of BubR1 knockdown. The results showed that suppression of BubR1 significantly decreased the percentage of migrated and invaded cells in KKU-055 and KKU-213A cells in the siBubR1 group compared to the scramble group, respectively (Figure 5A,B). In addition, Western blotting analysis of proteins related to epithelial–mesenchymal transition (EMT) that are involved in cell migration abilities showed increased expression of the epithelial marker zonula occludens-1 (ZO-1) and decreased expressions of mesenchymal markers including vimentin and slug in both CCA cell lines (Figure 5C,D).

### 3.6. Synergistic Effects of siBuR1 and Chemotherapeutic Drugs in CCA Cell Lines

We investigated the cytotoxic effects of siBubR1 combined with common chemotherapeutic drugs, including gemcitabine, cisplatin, and 5-fluorouracil, in CCA cell lines. Scramble and siBubR1 were treated in CCA cells for 24 h, and then the chemotherapeutic drugs were added and incubated for 48 h. The synergistic effects of siBubR1 and chemotherapeutic drugs are shown in Table 4. The combination index (CI) values were lower than 0.80, which indicated synergistic effects.

## 4. Discussion

Our study investigated the correlation between BubR1 expression and cholangiocarcinogenesis in hamster tissues. Results showed that BubR1 was slightly expressed in normal bile ducts but gradually increased in hyperplasia/dysplasia in OV-infected and NDMA-treated groups. This is the first report of BubR1 expression being up-regulated during cholangiocarcinoma in a hamster model. These findings suggested that BubR1 is involved in cholangiocarcinogenesis and could be detected early in precancerous CCA. mRNA and protein expression of BubR1 showed overexpression in CCA tissues compared to normal tissues, and patients with CCA with high BubR1 expression had a significantly shorter survival time. BubR1 was found to be an independent prognostic factor for the short survival of patients with CCA. These findings support earlier reports in liver [16], lung [10], and prostate [17] cancer.

BubR1 and MPS1 are important proteins in the spindle assembly checkpoint during mitosis, and are associated with the progression of cancer reported in lung adenocarcinoma (LUAD) [18]. According to recent research, the expression of MPS1 is a crucial predictor of a poor prognosis for patients with CCA [11]. Additionally, this research showed a notable link between BubR1 and MPS1 expression at the mRNA level. Interestingly, the study also discovered that high expression of both BubR1 and MPS1 was a more substantial independent prognostic factor for a short survival rate in patients with CCA as compared to the high expression of either BubR1 or MPS1 alone. This emphasizes the significance of studying the interaction between these two genes and their impact on the prognosis of CCA.

BubR1 expression was suppressed by siRNA, resulting in significantly reduced cell proliferation and colony formation, migration, and invasion, which was similar to previous reports on bile duct cancer [7] and lung cancer [10]. An increase in apoptotic cell death upon knockdown of BubR1 has previously been reported in liver cancer, resulting in cleaved caspase 3, and Bax was elevated, but Bcl-2 was reduced [8], and in thyroid cancer, caspase 3, 7, and 9 were increased [9]. Our study demonstrated that siBubR1 had an apoptotic effect via inducing cleaved PARP and reducing Mcl-1. Epithelial-to-mesenchymal transition (EMT) is a key step in cancer metastasis. Suppression of BubR1 promotes EMT by up-regulating the expression of epithelial markers (E-cadherin) and downregulating the expression of mesenchymal markers (N-cadherin and vimentin) [8]. Our findings showed for the first time that BubR1 suppression increased epithelial marker (ZO-1) expression while reducing mesenchymal marker (slug) expression.

A standard chemotherapeutic drug remains applied in clinical treatments for patients with CCA, whether or not using combined drugs to provide the treatment more effectively. However, the main problem for patients with CCA with advanced cholangiocarcinoma (CCA) is their resistance to available chemotherapy [19]. Komura and colleagues performed a comprehensive proteomic analysis and reported that BubR1 is the most up-regulated protein in chemoradiation therapy (CRT)-recurrent tumors as compared with a primary treatment-naïve tumor in bladder cancer; CRT-resistant bladder cancer cell lines were treated with cisplatin, and the result showed increased colony formation and BubR1 expression when compared to parent cells [20]. Our study demonstrated that combining siBubR1 with chemotherapeutic drugs, including gemcitabine, cisplatin, and 5-fluorouracil, showed significantly decreased cell viability compared to their control in CCA cell lines. This result indicated a synergistic effect that reduced the concentration of chemotherapeutic drugs. This finding suggested that suppression of BubR1 using siRNA in combination with chemotherapeutic drugs might be an alternative strategy for CCA treatment.

## 5. Conclusions

Our research reveals the clinical significance of BubR1 promoting cholangiocarcinogenesis, and that co-expression of BubR1 and MPS1 is associated with a short survival of patients with CCA. Suppression of BubR1 significantly inhibited cell growth, migration, and invasion, while increasing apoptosis in CCA. In addition, siBubR1 could synergize the cytotoxic effect of three chemotherapeutic drugs. These findings indicated that BubR1 may be an alternative target for CCA treatment.

## Figures and Tables

**Table 1:** BubR1 expression in Ov-associated CCA in hamster tissue.

	1 M		n (%)	3 M		n (%)	6 M		n (%)
	Low	High		Low	High		Low	High	
NBD	27 (84)	5 (16)	32 (100)	26 (68)	12 (32)	38 (100)	21 (62)	13 (38)	34 (100)
HP/DP	2 (25)	6 (75)	8 (100)	2 (14)	12 (86)	14 (100)	1 (7)	13 (93)	14 (100)
CCA	-	-	-	0 (0)	5 (100)	5 (100)	0 (0)	5 (100)	5 (100)

All data are shown as the number of low BubR1 and high BubR1 cases. The numbers in parentheses show the percentage of cases. NBD, HP/DP, and CCA stand for normal bile duct, hyperplasia/dysplasia, and cholangiocarcinoma, respectively. (-) stands for not detected.

**Table 2:** Univariate and multivariate analysis findings of 189 patients with CCA.

Variable (n)	Univariate Analysis	Multivariate Analysis		
	*p*-Value	HR	95%CI	*p*-Value
**Age (187)**	0.787			
(ref. group < 56)				
**Gender (189)**	0.019	1.599	1.053–2.429	0.028
(ref. group Female)				
**Histological types (180)**	0.488			
(ref. group papillary)				
**Lymph node metastases (175)**	<0.001	2.552	1.662–3.918	<0.001
(ref. group N0)				
**Tumor stage (189)**				
(ref. group I–III)				
IVA	0.023	1.179	0.750–1.855	0.476
IVB	<0.001	1.796	0.855–3.773	0.122
**Tumor size (189)**	0.195			
(ref. group < 5)				
**BubR1 expression (189)**	0.034	1.568	1.075–2.287	0.020
(ref. group low BubR1)				

**Table 3:** Univariate and multivariate analysis findings of BubR1 and MPS1 in 152 patients with CCA.

Variable (n)	Univariate Analysis	Multivariate Analysis		
	*p*-Value	HR	95%CI	*p*-Value
**Age (150)**	0.423			
(ref. group < 56)				
**Gender (152)**	0.029	1.356	0.891–2.065	0.155
(ref. group Female)				
**Histological types (144)**	0.324			
(ref. group Papillary)				
**Lymph node metastases (140)**	<0.001	2.055	1.396–3.026	<0.001
(ref. group N0)				
**Tumor stage (152)**				
(ref. group I–III)				
IVA	0.002	1.555	1.005–2.405	0.047
IVB	<0.001	2.721	1.402–5.281	0.003
**Tumor size (152)**	0.067			
(ref. group < 4)				
**BubR1 and MPS1 expression (152)**				
(ref. group Low BubR1 and MPS1)				
High MPS1 but low BubR1	0.386			
High BubR1 but low MPS1	0.708			
High BubR1 and MPS1	<0.001	2.778	1.578–4.891	<0.001

**Table 4:** Combination index (CI) and dose reduction index (DRI) of gemcitabine, cisplatin, and 5-fluorouracil treatment in CCA cell lines.

Cell Lines	siBubR1 (pmole)	Gem (µM)	Fa ± SD	CI	DRI	Cis (µM)	Fa ± SD	CI	DRI	5-FU (µM)	Fa ± SD	CI	DRI
					Gem				Cis				5-FU
KKU-055	50	0.25	0.43 ± 0.04	0.81	1.25	0.5	0.47 ± 0.08	0.18	17.75	5	0.60 ± 0.04	0.08	80.42
KKU-213A	50	0.5	0.55 ± 0.05	0.25	46.73	1	0.51 ± 0.04	0.20	83.28	1	0.53 ± 0.02	0.27	9.34

Fa: fraction affected is the percentage inhibition of cell proliferation.

## Data Availability

The original contributions presented in the study are included in the article, further inquiries can be directed to the corresponding author/s.
